# Copper Oxide Nanorods: Potential Agents against Breast Cancer

**DOI:** 10.1021/acsabm.4c01700

**Published:** 2025-05-16

**Authors:** Giovanna C. N. B. Lôbo, Ana Luísa G. Silva, Karine B. Barros-Cordeiro, Raquel das Neves Almeida, Ingrid Gracielle Martins da Silva, Matheus Pereira Sales, Leonardo Giordano Paterno, Sônia N. Báo

**Affiliations:** † Department of Cell Biology, Institute of Biological Sciences, University of Brasília, Federal District, Brasília 70910-900, Brazil; ‡ Laboratory of Research on Polymers and Nanomaterials, Institute of Chemistry, University of Brasília, Federal District, Brasília 70910-900, Brazil

**Keywords:** biotechnology, metallic nanoparticles, breast cancer, biocompatible nanoparticles

## Abstract

Breast cancer is the second most prevalent type of cancer worldwide and the leading cause of cancer-related mortality among women. Despite substantial advancements in scientific research, cancer continues to pose a major challenge due to its high global incidence. While numerous nontargeted therapies have been developed, nanotechnology-based platforms are emerging as a promising future for cancer treatment. In this context, we investigated the in vitro antitumor activity of citrate-coated copper oxide nanorods (CuO-nr, aspect ratio ∼3) against breast cancer cells. The CuO-nr colloids demonstrated stability for over 120 days under ambient conditions, with an average hydrodynamic diameter of 107.1 ± 0.67 nm and a zeta potential of −23.8 ± 1.87 mV. Cell viability assays showed that CuO-nr were minimally harmful to nontumor fibroblast cells but highly cytotoxic to MCF-7 breast cancer cells. Flow cytometry analysis suggested that early apoptosis may be the primary mechanism of cell death induced by these nanoparticles. Furthermore, significant alterations in cell morphology were observed through scanning electron microscopy. These findings indicate that citrate-coated CuO-nr possess effective antitumor activity and hold promise as potential agents for targeted cancer therapy.

## Graphical abstract

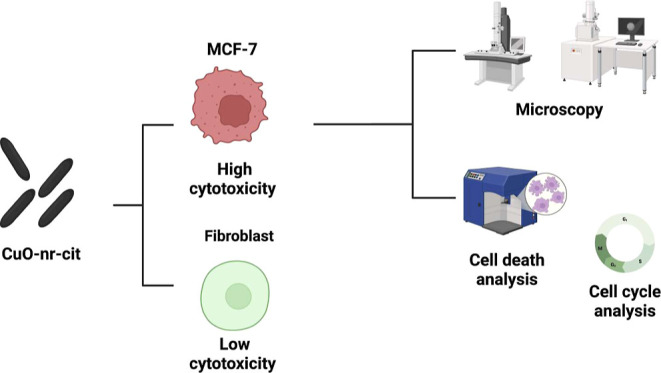

## 1 Introduction

Cancer encompasses a group of diseases characterized by the abnormal growth of cells.^1^ It ranks as one of the leading causes of death globally, second only to cardiovascular disease. Breast cancer is particularly significant as the second most common cancer worldwide and the leading cause of cancer-related mortality among women.^2^ This complex disease comprises over 20 different subtypes, with various factors—such as genetics, reproductive history, behavior, and lifestyle—closely linked to its development.^1–3^ The most recognized risk factors include aging, family history, breast density, oral contraceptive use, late menopause, hormone replacement therapy, and the early onset of menstruation. It is essential to recognize that these risk factors may evolve as new research and data emerge.^4,5^

As with other cancers, early diagnosis significantly impacts prognosis, leading to a higher survival rate of approximately 65% among patients. Diagnosis primarily occurs through regular examinations and mammography; however, the number of women receiving early diagnoses has been steadily declining each year.^6–9^

Conventional treatments typically include surgical resection, radiotherapy, chemotherapy, hormone therapy, and immunotherapy. Unfortunately, these approaches can damage healthy tissues and may not completely eradicate malignant cells, often resulting in a decreased quality of life for patients. Consequently, there is a pressing need to explore new therapeutic modalities that can enhance treatment efficacy and mitigate adverse effects.^10,11^

The medical revolution of this century has fostered interdisciplinary research, giving rise to an innovative field with promising biomedical applications: nanobiotechnology. This scientific discipline involves materials operating on atomic and molecular scales, exhibiting unique physical, chemical, and biological properties within a size range of 1 to 100 nm. Over the past decade, nanobiotechnology has facilitated the development of vital therapeutic tools, providing alternative solutions to the limitations in the diagnosis, treatment, and clinical management of human diseases.^12–14^

Among the diverse nanomaterials utilized for these purposes, metal and metal oxide nanoparticles play a crucial role in cancer treatment by enabling better targeting and drug delivery, thereby reducing the energy supply to tumors. They also serve as diagnostic tools for imaging cancer cells, facilitating simultaneous diagnoses and therapies, as well as controlled and targeted drug delivery.^15,16^ Copper oxide nanoparticles (CuO NPs), recognized for their exceptional thermophysical, electronic, and catalytic properties,^19–21^ are now being explored for biomedical applications. They exhibit biocidal activity^22^ and can prevent bacterial infections.^23^ Notably, they tend to accumulate in tumor tissues, following systemic administration, and can induce apoptosis in tumor cells, while elevating oxidative stress through the depletion of cellular antioxidants and mitochondrial dysfunction.^17,18^

Recent studies indicate that the oxidative stress-mediated toxicity of CuO NPs in various^24–28^ human cell lines can effectively induce apoptosis in tumor cells. Apoptosis induction is a critical mechanism for an effective anticancer agent, leading to significant morphological changes such as cell roundness or oval profiles, random distribution, cell shrinkage, and chromatin condensation. Conversely, other studies highlight the importance of necrosis in activating the immune response against cancer cells.^29^ These factors position CuO-nr as potential antitumor agents capable of inhibiting cancer cell proliferation.^30–32^

CuO-nr offers significant advantages over other nanoparticle forms, such as spheres, due to their larger surface area and greater number of active edges, enhancing catalytic efficiency and reagent molecule adsorption. This characteristic makes them particularly attractive for various applications in chemistry and biomedicine, including improved drug molecule adsorption and targeted delivery to cancer cells, thereby increasing treatment effectiveness.^33–36^ Thus, the present work proposes a novel platform for the therapy of breast tumor cell lines, utilizing citrate-coated copper oxide nanorods (CuO-nr-cit), which demonstrate potential in inducing tumor cell death.

## 2 Materials and Methods

### 2.1 Chemicals

Copper­(II) chloride dihydrate, 99% (CuCl_2_·2H_2_O), sodium hydroxide, 98% (NaOH), trisodium citrate dihydrate, 99% (Na_3_cit), DAPI, formvar solution, propidium iodide, and Leibovitz’s L-15 medium were bought from Sigma-Aldrich. Type-I water (resistivity: 18 Mohm cm) was supplied by a Millipore Milli-Q system. Breast cancer lines (MCF-7 and MDA-MB-231) and a nontumor line (MCF 10A and fibroblast) were acquired from the Rio de Janeiro Cell Bank (BCRJ). Dulbecco’s modified Eagle’s medium (DMEM), Roswell Park Memorial Institute medium 1640 (RPMI), fetal bovine serum (FBS), penicillin, and streptomycin were purchased from Gibco, Invitrogen (USA). AlamarBlueTM and Annexin-V-FITC came from Thermo Fisher. Osmium tetroxide and uranyl acetate came from Polysciences (USA). Spurr resin, sodium cacodylate, paraformaldehyde, glutaraldehyde 25%, and potassium ferricyanide were purchased from Electron Microscopy Sciences (USA).

### 2.2 Methods

#### 2.2.1 Elaboration and Structural Characterization of Citrate-Coated Copper Oxide Nanorods (CuO-nr-cit)

CuO-nr-cit were produced following the procedure described by Zhang et al., Mansano et al., and Waris et al.^26,37,38^ with slight modifications. The production of naked CuO-nr was conducted in a rounded-bottom, two-neck borosilicate glass flask connected to a glass condenser. About 75 mL of CuCl_2_·2H_2_O aqueous solution (0.02 M) was added to the above-described system and heated until boiling under vigorous magnetic stirring. Next, 0.4 g (0.01 mol) of NaOH was added at once, and the mixture was kept at the boiling point for another 15 min. The dark-brown precipitate of CuO-nr obtained after this period was isolated by decantation and submitted further to two cycles of washing with type-I water and centrifugation (8000 rpm) for 10 min, to remove the excess of unreacted chemicals. After that, the precipitate was suspended in Na_3_cit solution (1% w/w) under pulsed sonication (10 min, 1s on/1s off, 150 W) using a horn tip sonicator (Branson 450A). The resulting CuO-nr-cit suspension was transferred to a cellulose dialysis bag (molecular weight cutoff: 12 kDa) and dialyzed against double-distilled water for 48 h to remove the excess of citrate. Its concentration was determined by atomic absorption spectroscopy (Varian AA 240 FS) using a certified copper standard solution from Sigma-Aldrich. The aqueous colloid was digested in HNO_3_ 0.1 M solution, and aliquots of different concentrations were prepared for calibration. For some structural characterizations, an aliquot of CuO-nr-cit was lyophilized.

UV–vis absorption spectroscopy was carried out in a Varian Cary 5000 spectrophotometer (range: 200–800 nm; scan rate: 10 nm s^–1^; resolution: 0.5 nm). The Raman spectrum was recorded in a Raman inVia Renishaw microscope in backscattering configuration (range: 200–800 cm^–1^; excitation: 632.8 nm; laser power: 25 mW; resolution: 1 cm^–1^). X-ray diffraction was conducted with a D8 Focus Bruker diffractometer (increment: 0.05°; scan rate: 0.5° min^–1^). Thermogravimetric analysis was carried out with the DTG 60H Shimadzu thermoanalyzer (air atmosphere at 10 mL L^–1^; heating rate: 10 °C min^–1^). Transmission electron microscopy and selected area electron diffraction were performed with a JEOL JEM-2100 microscope operated at 200 keV. Colloidal properties (zeta potential and hydrodynamic size) were evaluated with a Malvern Zeta Sizer ZS90.

#### 2.2.2 Cytotoxicity Assay

Experiments were carried out using two breast cancer lines (MCF-7 and MDA-MB-231) and two nontumor lines (MCF 10A and fibroblasts), and the cells were cultured in RPMI, L-15, DMEM HIGH, and DMEM-F12, respectively. All media were supplemented with 10% fetal bovine serum (FBS) and 1% antibiotic (penicillin–streptomycin) and maintained at 37 °C in the presence of CO_2_, except for the MDA-MB-231 cell line, which does not need to be grown in CO_2_. Cell viability was determined using different concentrations of CuO-nr-cit (25; 50; 75; 100 μg mL^–1^) and a negative control (NT) based on bibliographic studies^24,29–36^ and with different times (24, 48, and 72 h). Cells were plated (3 × 10^4^ per well) by being seeded into 96-well cell culture plates and grown for 24 h at 37 °C. Afterward, cells were exposed to treatments for certain times. After incubation, a fresh medium containing 10% AlamarBlueTM reagent was added, in accordance with the datasheet (Thermo Fisher, USA). Cells were further incubated for 3 h at 37 °C. Fluorescence was measured using a SpectraMax M5 spectrophotometer (Molecular Devices, USA) (λex 560; λem 590). The inhibitory concentration value (IC50) and 95% confidence interval were derived from a nonlinear regression model based on the sigmoidal dose response curve (variable slope) and computed using GraphPad Prism 5.0 (CA, USA).

#### 2.2.3 Cell Morphology and Ultrastructural Analysis

Cell morphology assay was performed in accordance with Silva et al.^5^ Cell lines were visualized by the AxioVert light microscope (Zeiss, Germany) and images captured using the AxioVision 100 program, after treatment with CuO-nr-cit (50 μg/mL^–1^) for 3, 6, and 48 h. For ultrastructural analysis (SEM), 1 × 10^4^ cells were dispersed in 6-well plates. After the treatment (CuO-nr-cit at 50 μg mL^–1^ for 48 h), cells were washed with PBS and fixed overnight at 4 °C in solution containing 2% paraformaldehyde (v/v), 2% glutaraldehyde (v/v), and 0.1 M sodium cacodylate buffer pH 7.2. Afterward, cells were postfixed, for 30 min, in 2% osmium tetroxide (w/v) and 1.6% potassium ferricyanide (10 mM CaCl_2_ in 0.2 M sodium cacodylate buffer, pH 7.2). The material was dehydrated in a graded acetone series (50–100%) for 10 min each. Then, the samples were critical-point-dried (Balzers, CPD 030, Germany) from liquid CO_2_ and gold-sputtered (SCD 500, LEICA-Germany). Images were obtained by a JSM-7001F (Jeol Japan) scanning electron microscope (SEM). For ultrastructural analysis (TEM), 1 × 10^5^ cells were dispersed in 6-well plates. After the treatment (CuO-nr-cit at 50 μg mL-1 for 48 h), cells were washed with PBS and fixed overnight at 4 °C in a solution containing 2% paraformaldehyde (v/v), 2% glutaraldehyde (v/v), and 0.1 M sodium cacodylate buffer pH 7.2. Afterward, cells were postfixed, for 30 min, in 2% osmium tetroxide (w/v) and 1.6% potassium ferricyanide (10 mM CaCl_2_ in 0.2 M sodium cacodylate buffer, pH 7.2). Samples were washed in distilled water and put into 0.5% uranyl acetate for 24 h at 4 °C. The material was dehydrated in a graded acetone series (50–100%) for 10 min each and embedded in Spurr resin. Ultrathin sections were obtained from an ultramicrotome UC6 (Leica, Germany) and observed in a Jeol 1011 transmission electron microscope (TEM) at 80 kV.

#### 2.2.4 Cell Death by Annexin-V/Propidium Iodide Staining Analysis

Cells (2 × 10^5^ per well) were dispersed in 6-well plates and incubated with the CuO-nr-cit (CuO-nr-cit at 50 μg mL^–1^ for 48 h). After the treatment, cells were washed with PBS and resuspended in a solution containing 100 μL of blocking buffer (10 mM HEPES/NaOH (pH 7.4), 140 mM NaCl and 2.5 mM CaCl_2_), 3 μL of Annexin-V-Alexa 488 (Invitrogen, USA), and propidium iodide (5 μg mL^–1^) (Molecular Probes, USA). In the next step, the cells were incubated for 15 min, in the dark, at room temperature. Subsequently, 400 μL of PBS buffer was added, and 10,000 events of each sample were acquired by a FACSCalibur flow cytometer, channels FL-1 and FL-4 (Becton Dickinson, USA), with the aid of CellQuest-Pro software.

#### 2.2.5 Cell Cycle Analysis by Propidium Iodide (PI) Labeling

To evaluate and quantify the cell populations that were in G0/G1 (2N DNA), in G2/M (4N of DNA), and in S phase (intermediate amount of DNA), an assay was performed to carry out DNA labeling with PI and flow cytometer analysis, in accordance with the protocol described in da Rocha et al.^41^ The cells (3 × 10^5^) were plated in 12-well plates, and after 24 h, they were treated with the culture medium (control) or with CuO-nr-cit. After 24 h, the cells were detached and centrifuged at 300*g* for 3 min to remove the culture medium. The pellet was washed in 500 μL of 1× PBS, 4.5 mL of 70% ethanol, and kept for 2 h on ice for fixation. Subsequently, the cells were washed with PBS and incubated in PI solution (0.1% triton X-100, 10 μg/mL PI and 100 μg/mL DNase-free) diluted in PBS for 10 min at room temperature. After incubation, the samples underwent a centrifugation process and were resuspended in 1× PBS, and 8000 events from each sample were analyzed by flow cytometry. The DNA content of cells in the different phases of the cycle was determined by the cell flow cycle platform FlowJo software. Three independent experiments were performed.

#### 2.2.6 Statistical Analysis

All the data obtained were analyzed in the GraphPad Prism 5.0 (CA, USA) program and then submitted to specific tests with a statistical confidence of 95%. Two-way ANOVA tests and Tukey post-tests were used. Data were presented as mean value ± SEM of at least three independent experiments. The significance level was defined as *p < 0.005, **p < 0.01, ***p < 0.001, and ****p < 0.0001.

## 3 Results and Discussion

### 3.1 Synthesis and Characterization of Copper Oxide Nanorods Surface-Coated with Citrate

The physicochemical characteristics of plain and citrate-coated copper oxide nanorods, CuO-nr and CuO-nr-cit, were evaluated by a set of analytical methods: X-ray diffractometry (XRD), UV–vis absorption, Fourier-transform infrared (FTIR) and Raman spectroscopies, thermogravimetric analysis (TGA), and high-resolution transmission electron microscopy (TEM). Besides that, the colloidal properties of the CuO-nr-cit aqueous suspension were determined by dynamic and electrophoretic light scattering. The main results are collected in Figure 1.

**Figure 1:**
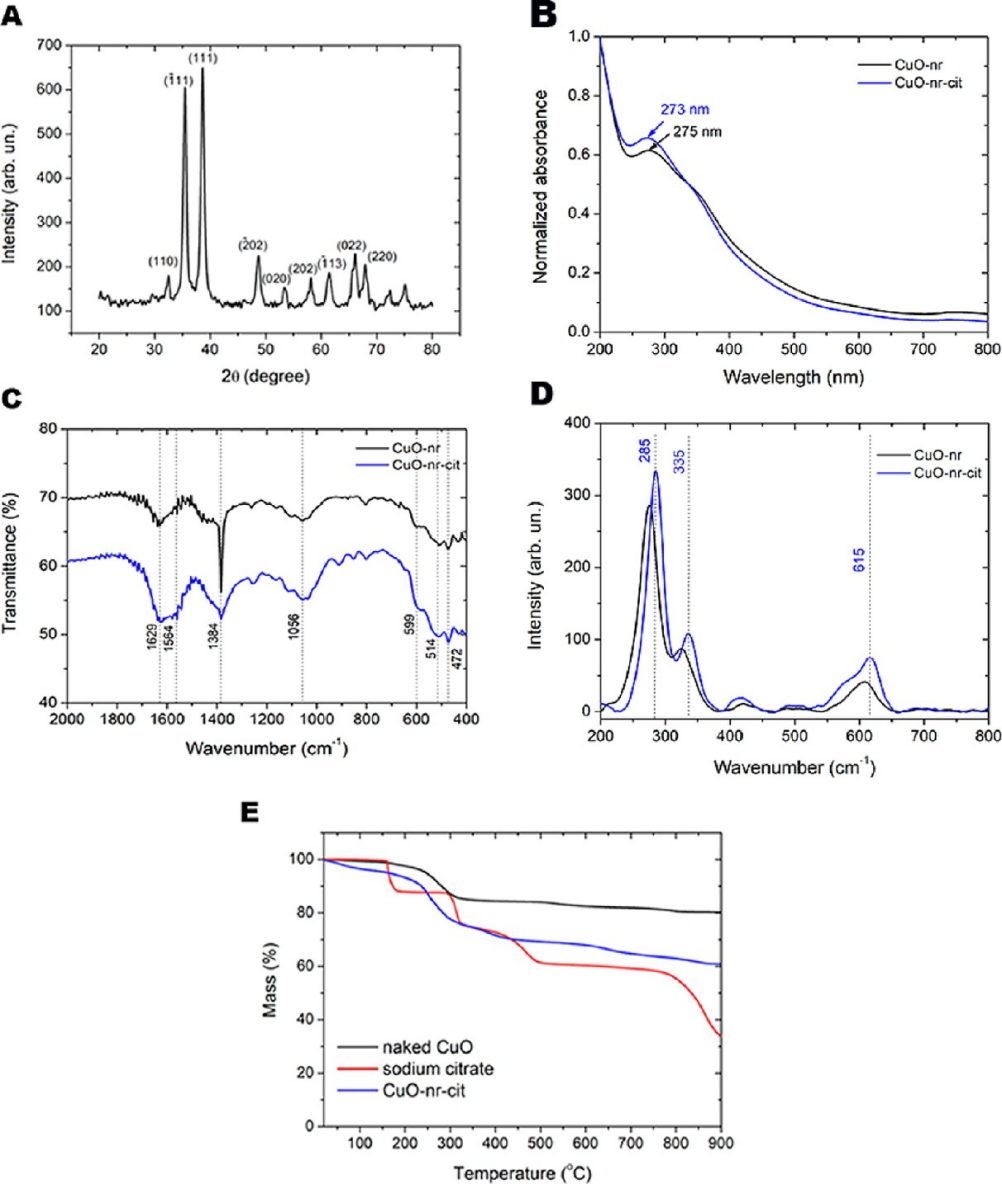
CuO-nr and CuO-nr-cit characterization. (A) X-ray diffractogram, (B) UV–vis absorption spectrum, (C) FTIR spectrum, (D) Raman spectrum, and (E) TGA curves.

The X-ray diffractogram (Figure 1A) indicated the crystalline structure of CuO in the monoclinic phase.^34^ The mean crystallite diameter (*D*_*hkl*_) was determined using the Scherrer equation (eq 1) and the full width at half-maximum (fwhm) of the most intense diffraction peak, (110), which was obtained after proper Lorentzian adjustment. For λ = 1.54 Å (wavelength of the X-ray beam radiation source (Cu Kα)), the *D*_*hkl*_ found was 48 nm.

1 $$D_{hkl}=\frac{0.9\lambda }{\beta \cos\theta }$$

The UV–vis spectra (Figure 1B) of CuO-nr and CuO-nr-cit appear quite similar, with a maximum absorption centered at 275 and 273 nm, respectively, which is attributed to a direct band gap transition in CuO.^29,30^ A second and more subtle absorption, also assigned to a direct band gap transition, is seen around 345 nm for both materials, although this is less evident in CuO-nr-cit. Although small, these changes are due to functionalization of the nanoparticles with citrate.^31^

Figures 1C and 1D show the vibrational structure of the nanoparticles in IR and Raman modes, respectively. Since monoclinic CuO belongs to the *C*2/*c* space group, group theory predicts 12 phonon modes for it or else F047vib = Ag + 2Bg + 4Au + 5Bu. Six of them are IR-active, 3Au + 3Bu, whereas three, Ag + 2Bg, are Raman-active. The FTIR spectra (Figure 1C) of plain CuO-nr and CuO-nr-cit exhibit three vibration modes at 472 cm^–1^ (Bu), 514 cm^–1^ (Bu), and 599 cm^–1^ (Au), which are ascribed to stretching of the Cu–O bond along [1 ®01] and [101] directions.^32^ In addition, the spectra display other vibrational modes at 1629 cm^–1^, 1384 cm^–1^, and 1056 cm^–1^, which are associated with O–H stretching in adsorbed water and some remaining Cu–OH groups. In the spectrum of CuO-nr-cit, there is also a broad peak at 1564 cm^–1^, which is assigned to the −COO– asymmetric stretching due to citrate molecules coating the nanoparticle. The Raman spectra (Figure 1D) of both samples exhibit the expected vibrational modes, namely, A_g_ (∼280 cm^–1^), Bg1 (∼330 cm^–1^), and Bg2 (∼615 cm^–1^), which may undergo deviations according to local lattice strain and adsorbed species.^33–35^ Indeed, in the spectrum of CuO-nr-cit (in blue), they are all blue-shifted by about 10 cm^–1^, except for the Bg2 mode, which is shifted by only 6 cm^–1^. The shift reveals a stronger interaction of phonons with the incident radiation, possibly caused by a compressive stress of weakly bonded surface atoms of nanoparticles in the presence of citrate molecules.

Additionally, Figure 1E shows TGA curves for sodium citrate, CuO-nr, and CuO-nr-cit. The main difference between CuO-nr and CuO-nr-cit is the residual mass at the end of the experiment, which is lower in the latter. This is because part of its mass is due to citrate molecules, which are decomposed upon heating. In fact, citrate makes up about 24% of the mass of CuO-nr-cit, as determined by the difference between the two curves.

The histograms shown in Figure 2A,B provide the mean length and mean width of CuO-nr-cit obtained after counting ∼300 particles in different TEM images. The average sizes, obtained after log-normal adjustment, were equivalent to 41.45 ± 0.47 nm and 13.99 ± 0.1 nm, for length and width, respectively. It is noteworthy that the length of the nanoparticle is compatible with the size of the crystallite determined by X-ray diffraction (48 nm).

**Figure 2:**
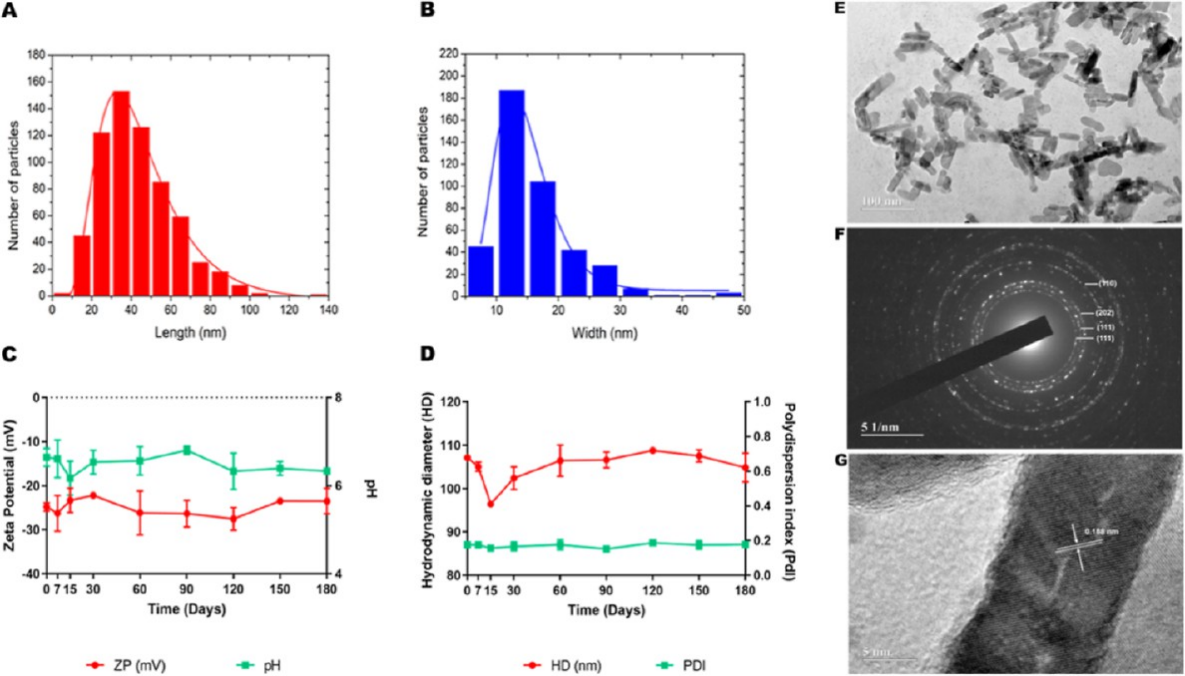
CuO-nr and CuO-nr-cit ultrastructure and stability over time. (A) Length and (B) width. The average sizes, obtained after log-normal adjustment, were equivalent to 41.45 ± 0.47 nm and 13.99 ± 0.31 nm, for length and width, respectively. Monitoring of the colloidal stability of CuO-nr-cit for 180 days; (C) zeta potential (mV) and pH; (D) hydrodynamic diameter (HD) and polydispersity index (PdI); (E) TEM image. Scale bar = 100 nm. (F) Selected area electron diffraction pattern and (G) high-resolution TEM image. Scale bar = 5 nm. Size distribution histograms for CuO-nr-cit.

Figure 2C,D shows the evolution of the colloidal properties of CuO-nr-cit in terms of surface charge (zeta potential) and pH (Figure 2C), as well as hydrodynamic diameter and polydispersity index (Figure 2D). Over 180 days, the zeta potential varied between −30 mV and −20 mV and the pH between 7 and 6. The hydrodynamic diameter showed significant variations on the first days of monitoring, decreasing until the 15th day and returning to the initial value (107.1 ± 0.67 nm) thereafter. Visual inspection, carried out periodically, did not indicate the formation of sediments or flakes, and based on the results shown in Figure 1, it was concluded that the colloidal suspension of CuO-nr-cit was stable, at least during the monitoring period.

The CuO-nr-cit was also characterized by high-resolution TEM and, as shown in Figure 2E, the nanorods have a typical rod morphology with faceted tips. The selected area electron diffraction pattern (Figure 2F) was indexed with the most intense diffraction planes in the CuO monoclinic phase according to the previous X-ray diffraction pattern (Figure 2E). The high-resolution micrograph in Figure 2G reveals crystallographic planes in detail. The estimated mean interplanar distance from this micrograph is 0.188 ± 0.10 nm, which corresponds to the $\left(\underline{\overset{®}{2}}00\right)$ plane after using the Bragg equation (eq 2), where *n* is an integer, *d* is the interplanar distance of the crystal plane, and λ and θ correspond to the wavelength and angle of diffraction, respectively.

nλ=2dsen(θ) 2

### 3.2 Cell Viability Studies

For the application of the developed formulation in cancer therapy, as an important step, the in vitro cytotoxicity of CuO-nr-cit and free copper ion (Cu^2+^) was evaluated. In this work, this assay was performed in two breast carcinoma cell lines (MDA-MB-231 and MCF-7) (Figure 3A,B) and in a nontumor cell line (MCF 10A) (Figure 3C). The results suggest that CuO-nr-cit showed dose- and time-dependent toxicity in the MCF-7 cell line compared to NT (negative control). The tumor cell line (MCF-7) (Figure 3A) showed more cytotoxicity by the nanostructure, showing a significant reduction in viability at higher concentrations of CuO-nr-cit (75–100 μg mL^–1^). The MDA-MB-231 cell line (Figure 3B) responded to treatment (∼50%), demonstrating that it was more resistant to the proposed therapy, showing an improvement in cell viability even after exposure of the nanostructure at the highest dose and longest time. The nontumor cell line (MCF 10A) (Figure 3C) was also more sensitive (∼60%) to CuO-nr-cit treatment, showing a significant reduction in viability. However, fibroblasts, the other nontumor cell line, showed more resistance to CuO-nr-cit, demonstrating a maximum reduction of 30% of viability after treatment (Figure 3D). Indeed, that could indicate that CuO-nr-cit did not exhibit cytotoxicity in healthy cells. In the scientific literature, it has been observed that metal oxide-based nanoparticles do not cause high toxicity in cultured cells with a concentration below 50 μg/mL^–1^, a fact that corroborates the results found after tests with CuO-nr-cit performed in the present work.^37,38^

**Figure 3:**
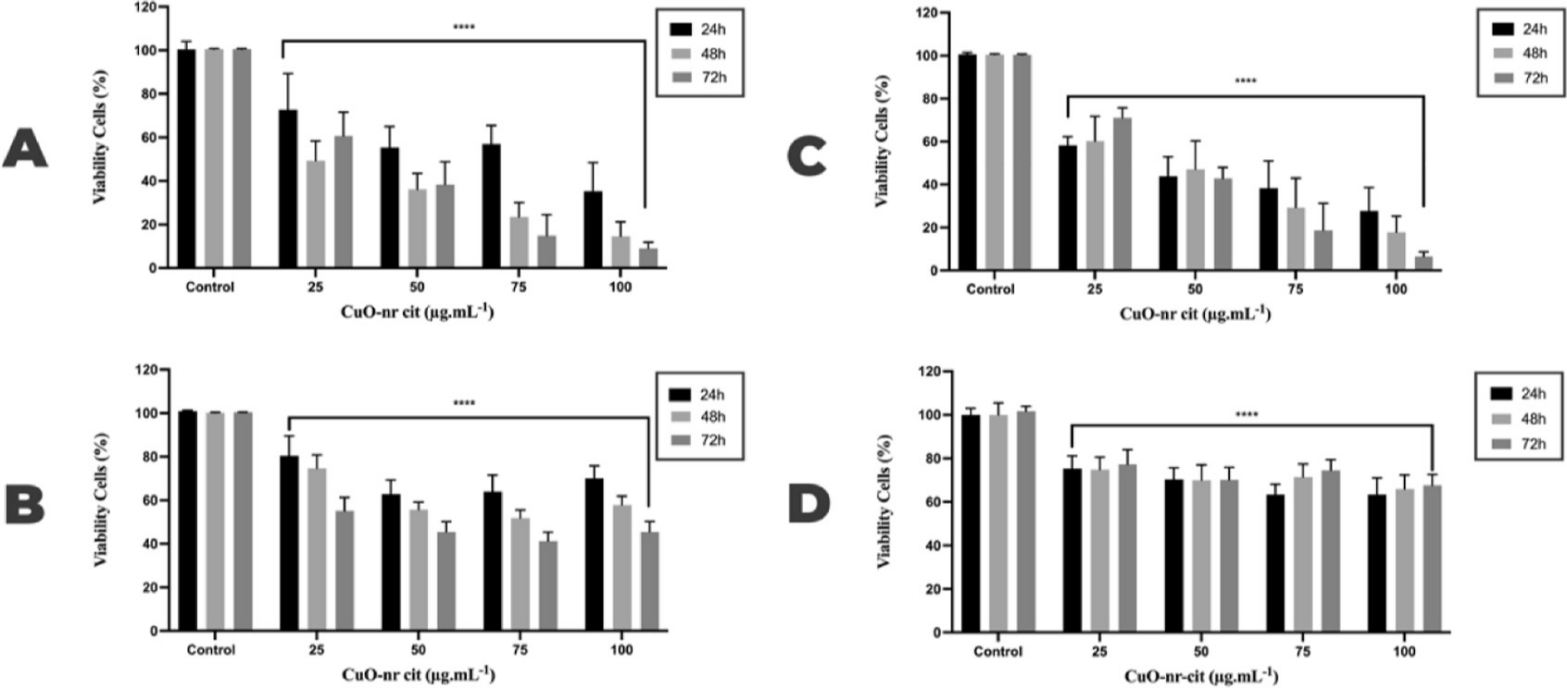
Evaluation of cell viability by alamarBlueTM of MCF-7, MDA-MB-231, and MCF 10A strains, and fibroblasts after 24, 48, and 72 h of treatment with CuO-nr-cit at different concentrations. Viability chart of strain (A) MCF-7, (B) MDA-MB-231, (C) MCF 10A, and (D) fibroblasts. Bars represent percentage cell viability for each strain at the indicated concentrations. Data represent the mean ± SEM of three independent experiments in quadruplicates ****P < 0.0001. Treatment compared to untreated control.

The in vitro cytotoxicity of free Cu^2+^ ions was also assessed to determine if the compound alone would induce toxicity in both tumor and nontumor cell lines. The tumor cell line MCF-7 (Supporting Information S1, Figure S1A) exhibited a significantly greater reduction in viability (∼60%) across the four established concentrations when compared to CuO-nr-cit, indicating that Cu^2+^ in its free form is more cytotoxic to this tumor lineage than when associated with the nanoparticle. Similarly, the MDA-MB-231 cell line (Supporting Information S1, Figure 5B) displayed a notable decrease in viability (∼40%) compared to CuO-nr-cit, further highlighting this line’s resistance to nanoparticle treatment. In contrast, the nontumor cell line MCF 10A (Supporting Information S1, Figure 5C) showed increased sensitivity to Cu^2+^ treatment, particularly at concentrations above 50 μg/mL, compared to the nanosystem, suggesting a heightened vulnerability to the free ion.

The 50% inhibitory concentration (IC_50_) is commonly used in fundamental aspects of pharmacology.^36–38^ This value refers to the concentration that results in 50% cell death in the proposed treatment. In this experiment, these rates were calculated for all treatment periods performed with the two cell lines. For the MDA-MB-231 tumor cell, it was not possible to calculate the IC_50_ score for the cell viability at any of the treated times, as the concentrations used did not reduce cell viability by 50%. As shown in Table 1, when comparing the IC_50_ values, we observed that a higher concentration was required to reduce the viability of MCF-7 tumor cells by 50%, compared to the nontumor MCF 10A cells. This is a particularly interesting finding, as it highlights that in therapeutic contexts, the IC_50_ concentration effective for MCF-7 cells would not result in significant cell death in MCF 10A cells. This suggests a potential for selective cytotoxicity, where tumor cells can be targeted while minimizing damage to healthy cells.

**Table 1:** Inhibitory Concentration of 50% CuO-nr-cit of Strains MCF-7 and MCF 10A at 24, 48, and 72 h

time	24 h	48 h	72 h
MCF-7	35.97 μg mL^–1^	33.85 μg mL^–1^	16.63 μg mL^–1^
MCF 10A	28.82 μg mL^–1^	35.3 μg mL^–1^	38.31 μg mL^–1^

The values obtained by calculating the IC_50_ after treatment with copper (Cu) reveal that for the MCF-7 cell line, the inhibitory concentration is higher when compared to the results analyzed for CuO-nr-cit (Supporting Information S2); on the other hand, for MCF 10A, they show increased values of IC_50_ for free Cu^2+^ when compared to the values obtained with the nanostructure.

### 3.3 Morphological and Structural Changes in Tumor and Nontumor Cell Lines

The CuO-nr nanostructure was able to induce morphological alterations in the studied tumor cell lines (MCF-7 and MDA-MB-231). Images obtained by light microscopy, in Figure 4, show that a decrease in the number of adhered cells can be observed when compared to the nontumor cell lineage (MCF 10A). In addition, the tumor cell line underwent more significant morphological changes, including reduction in cell size, loss of adhesion focal points, and characteristic cytoplasmic projections, especially after 48 h, suggesting death and/or interruption of the cell cycle caused after treatment. The nontumor cell line (MCF 10A) underwent little loss of quantity and points of adhesion, demonstrating fewer alterations in cell morphology than the tumor cell lines (Figure 4).^39,40^

**Figure 4:**
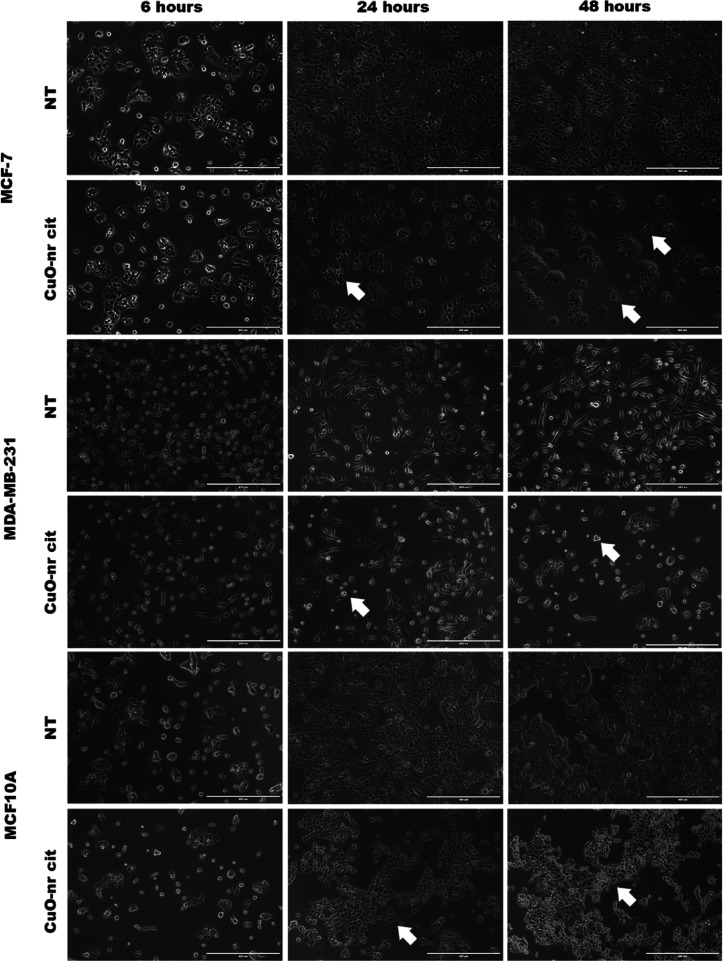
Evaluation of cell morphology of tumor (MCF-7 and MDA-MB-231) and nontumor (MCF 10A) cell lines after 6, 24, and 48 h of exposure to CuO-nr-cit treatment (50 μg/mL^–1^). Light Microscopy Images (phase contrast). NT (untreated cells) and cells treated with CuO-nr-cit after 6, 24, and 48 h (50 μg/mL^–1^). Arrows indicate decrease in adhered cells and cell size. Scale bar = 20 μm.

As in other studies, to evaluate the effect of CuO-nr-cit on cell morphology, scanning electron microscopy (SEM) was performed (Figure 5).^5,41–43^ Similar to phase contrast light microscopy, this indicated a reduction in tumor cell volume and adhesion points. The images allow the observation of morphological changes in cell surfaces, which presented a more rounded and rough appearance after treatment compared with the control. Figure 5 shows that MCF-7 and MDA-MB-231 tumor cells decreased in size in addition to acquiring a rougher appearance on their surface. In the nontumor cell line MCF 10A, cell morphology changed and the surface of untreated control cells became rougher. However, cells whose size and surface area were unchanged compared to tumor cells were also observed.

**Figure 5:**
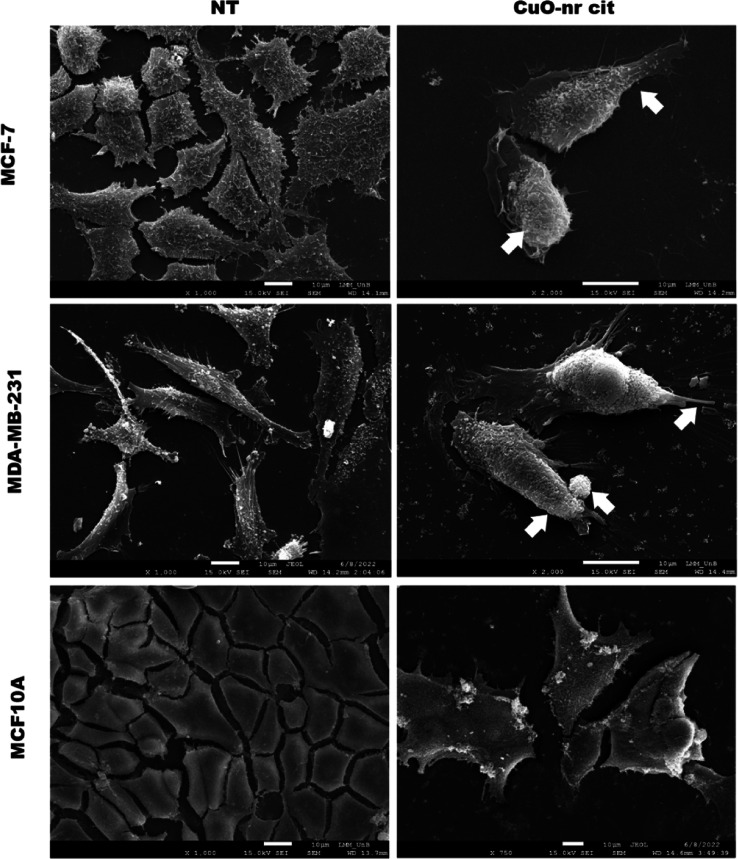
Change in morphology of tumor and nontumor cell lines after exposure to treatment with CuO-nr-cit. Scanning electron microscopy (SEM). NT (untreated cells) and cells treated with CuO-nr-cit after 48 h (50 μg/mL^–1^). White arrows indicate cytoplasmic processes. Scale bar = 10 μm.

### 3.4 Cell Death Profile

Understanding the nature of cell death is crucial, as it can elucidate the mechanisms by which nanostructures interact with the studied cell line.^44–47^ To investigate the type of cell death induced by CuO-nr-cit, a concentration of 50 μg/mL^–1^ was employed. After an incubation period of 48 h, the cells were stained with Annexin V and propidium iodide (PI), which are markers used to evaluate subpopulations of viable cells, apoptotic cells, and lytic cell death (Figure 6A). The destruction of tumor cells via the induction of apoptosis and necrosis is a significant consideration when integrating immune cells into antitumor therapies.^48,49^

An interesting finding was that treatment with CuO-nr-cit in MCF-7 cells induced lytic cell death (∼70%) (Figure 6B), while in MCF10A cells, despite a 60% reduction in viability, the cell death triggered by the treatment was apoptotic. This phenomenon suggests that nanoparticle treatment may exert a cytolytic effect on tumor cells but not on nontumor cells, indicating a potential pharmacological benefit. Additionally, in fibroblasts, there was no change in cell viability after treatment with CuO-nr-cit, demonstrating that the cytotoxic effect of the nanoparticles is specific to MCF-7 cells.

**Figure 6:**
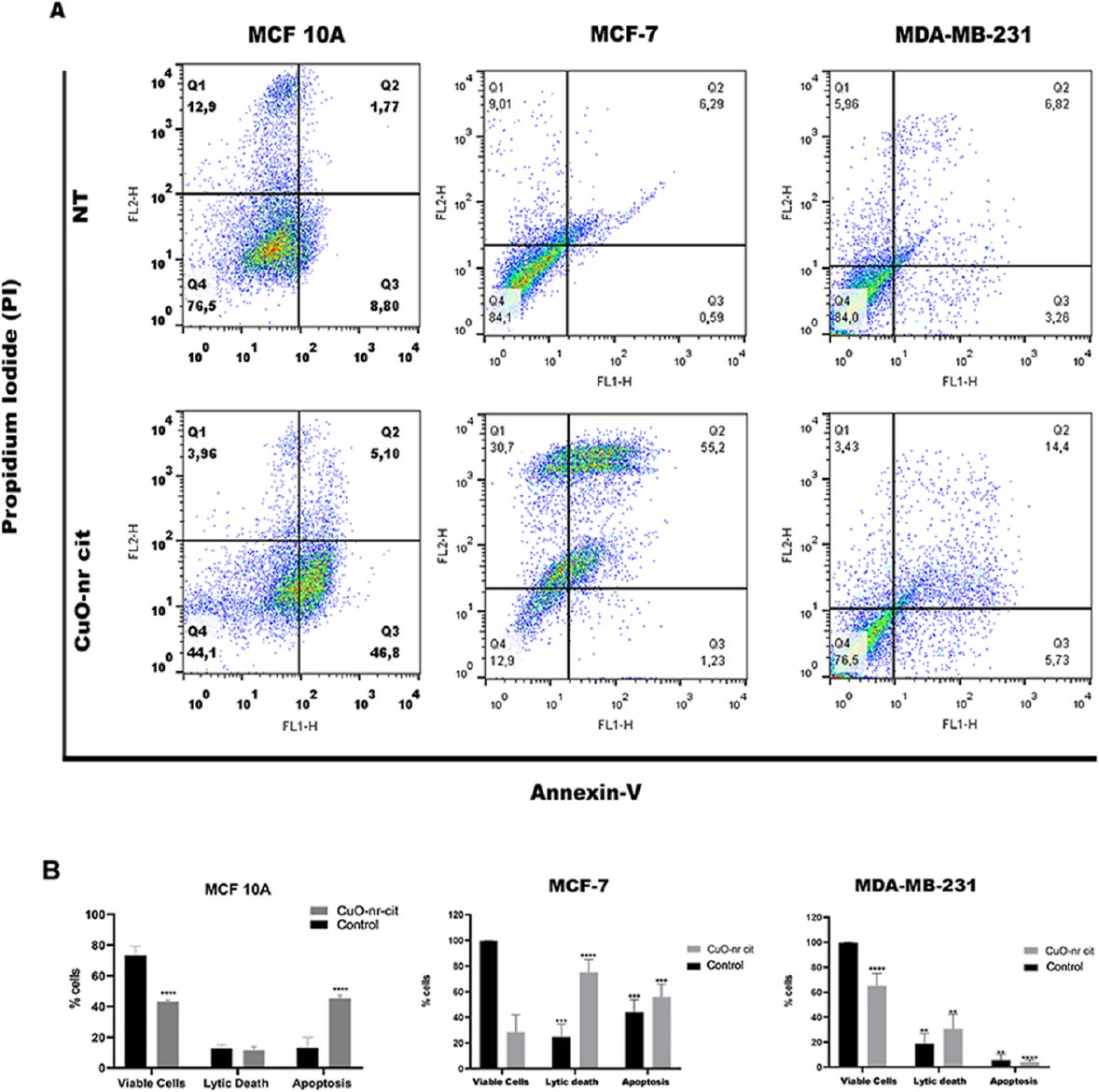
Evaluation of the cell death profile induced by CuO-nr-cit (50 μg/mL^–1^, 48 h) in MCF 10A, MCF-7, and MDA-MB-231 cells. (A) Distribution of cell populations according to the marking profile. Lower left quadrant corresponds to viable cells, lower right to apoptotic cells, upper left to necrotic cells, and upper right to lytic cell death. (B) Graphical representation of the cell death profile with statistical analysis. **P < 0.01; ***P < 0.001 and ***P < 0.0001. Compared to untreated control.

The MDA-MB-231 strain exhibited resistance to the treatment, with only approximately 14.5% of cells undergoing lytic death and 3.43% experiencing necrotic death (Figure 6B). The observation of cell death via both apoptosis and necrosis underscores the need for further studies to explore how this nanostructure may contribute to immune system activation, a process that is vital for effective cancer therapy.^44,46–56^

### 3.5 Cell Cycle Analysis

To assess the influence of CuO-nr on cell cycle progression, the cells were treated for 48 h with an IC_50_ dose and, after this period, they were marked with propidium iodide (PI) for analysis in a flow cytometer. The DNA content of the population was observed by marking with propidium iodide (PI). This marker is a DNA intercalator, and therefore the amount of marking present in the cell is related to the amount of DNA that the cell has. As with flow cytometry analysis, it is possible to quantify cells in G0/G1; with 4N DNA, they could be G2/M; the mutants, which are in the S phase, can be quantified too. Furthermore, those cells whose DNA is fragmented present a fluorescence quantification below the G0/G1 peak, being then called the sub-G1 peak.^57^

The results obtained through this analysis proved that for the MCF-7 tumor lineage, the treatment with CuO-nr-cit significantly alters the G2/M, which had its acceleration increased in relation to the untreated control. Abdel-Ghany et al.^57^ showed that AuNPs were situated within MCF-7 cells near the nucleus, resulting in cytotoxic effects by arresting the cell cycle in the G2/M phase. The MDA-MB-231 cell did not show alterations in the cell cycle profile when detected in the control (Figure 7).

**Figure 7:**
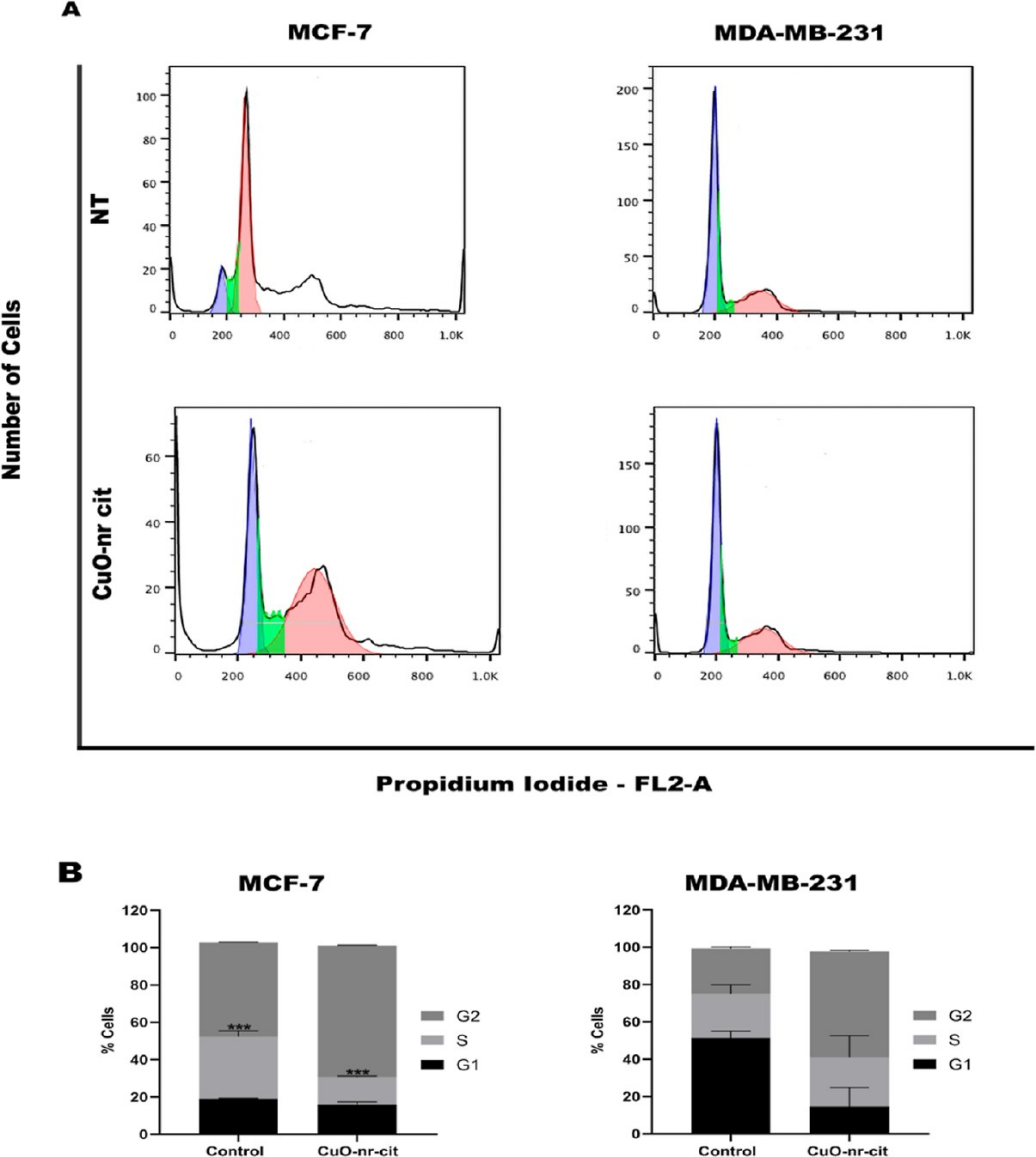
Cell cycle assessment in MCF-7 and MDA-MB-231 cells treated with CuO-nr cit (IC50, 48 h). (A) Representative histogram of the cell cycle phases pre- and post-treatment with the nanostructure. The histogram cores represent the phases of the cell cycle, blue for the G1 phase, green for the S phase, and red for the G2 phase. (B) Percent quantification of cells in each phase of the cell cycle. Data represent three independent experiments, *n* = 1, expressed as mean percentage of cells in each cell cycle phase ± SEM ***P < 0.001.

### 3.6 Internalization of CuO-nr-cit Evaluated by TEM

Transmission electron microscopy was performed to analyze the internalization of CuO-nr-cit. According to Loufty et al., 2015, Gupta et al., 2022, and Jarrar et al., 2023,^58–60^ the level of electron density is associated with the internalization of metallic nanostructures, which is observed after 3 and 48 h of treatment in both cell lines (Figure 8). In MCF-7 cells, more electron-dense structures were observed around the lysosomes and in the peripheral regions, suggesting the presence of nanostructures after 48 h of treatment. Dispersed clusters were also noted in the cytoplasm, especially when compared to those of untreated cells. Additionally, after 3 h of treatment, the presence of the autophagic vacuoles was observed in both cell lines, which can indicate the initial phase of cell death. Notably, the presence of nanoparticles in the cell nucleus was not observed.

**Figure 8:**
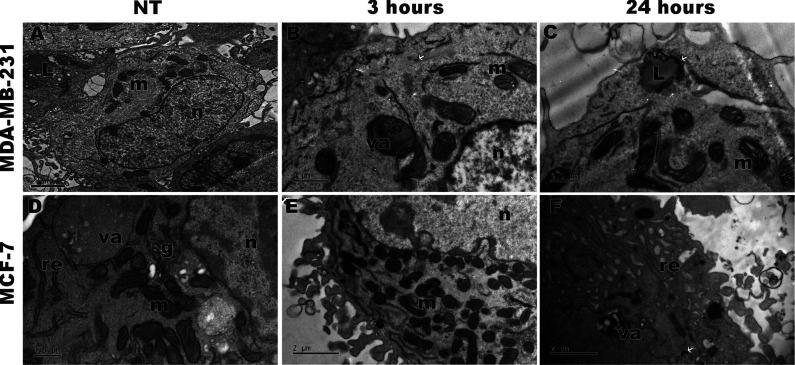
Evaluation of the internalization of CuO-nr-cit nanoparticles in MCF-7 and MDA-MB-231 tumor cell lines. Transmission Electron Microscopy (TEM). (A) MCF-7 control and (B) MCF-7 after 3 h of treatment, (C) MCF-7 after 48 h of treatment, (D) MDA-MB-231 control, (E) MDA-MB-231 after 3 h of treatment, and (F) MDA-MB-231 after 48 h of treatment. *N* = nucleus, L = lysosomes, re = endoplasmic reticulum, g = Golgi complex, va = autophagic vacuoles, and the white arrows indicate the internalized nanoparticles.

## 4 Conclusions

In conclusion, this study demonstrated the great potential of copper oxide nanorods as a therapeutic agent against breast cancer, particularly for the MCF-7 tumor cell line. Like some other studies reported in the literature, copper presents several advantages in biomedical applications, including its ability to induce oxidative stress, compromising mitochondrial integrity and activating the immune system response and inflammation.^21,59,61,62^ On the other hand, the nanorods’ shape offers significant advantages in the therapeutic context, such as increased surface area and active edges that enhance interactions with target cells, improving catalytic efficiency, promoting internalization, and reducing the typical effects of conventional therapies.^21,63^ In our study, CuO-nr-cit shows selective cytotoxicity, inducing greater cytotoxicity in the MCF-7 cell line while exhibiting low toxicity in nontumor cells, like fibroblasts and MCF10A, especially at lower concentrations. The results of a study with a breast cell line (MCF10A) showed that this cell line was more sensitive to CuO-nr-cit, a fact that can be explained by the evidence even in its culture method, requiring specific conditions to ensure its viability and growth, which are reflected in the intrinsic vulnerability and the increase in cytotoxicity.^64,65^ Flow cytometry assays reinforced the efficacy of copper oxide nanorods, showing lytic cell death in MCF-7 tumor cells. This offers a significant advantage for future studies aiming to improve cancer immunotherapy, given that this type of cell death could produce a possible local inflammatory response, leading to the recruitment and activation of the immune system.^61,62^ Finally, we demonstrate that the CuO-nr-cit nanosystem shows a potential application in drug delivery systems, and the nanorods’ shape can assist in the induction of cytotoxicity. In the future, it is essential to further explore the interaction of copper nanorods with the immune system, particularly regarding the activation of antitumor responses. Additionally, more studies could focus on overcoming the resistance observed in MDA-MB-231.

## Supplementary Material



## Data Availability

The data sets used and/or analyzed during the study are available from the corresponding author on reasonable request.

## Notes

AlamarBlue cell viability assay data for MCF-7, MDA-MB-231, and MCF 10A cell lines and IC_50_ values for free Cu^2+^ at 24, 48, and 72 h (PDF)
