# Supplementary material for: Copper Oxide Nanorods: Potential Agents against Breast Cancer
Source: ACS Appl Bio Mater. 2025 May 16;8(6):4621–32. doi: 10.1021/acsabm.4c01700 (PMC12175131; doi:10.1021/acsabm.4c01700)
Supplement: Supplementary file 1 [file mt4c01700_si_001.pdf]

Supporting Information

Copper oxide nanorods: Potential agents against breast cancer.

Giovanna C. N. B. Lôbo[1], Ana Luísa G. Silva[1], Karine B. Barros-Cordeiro[1], Raquel das Neves Almeida[1], Ingrid Gracielle Martins da Silva[1], Matheus Pereira Sales[2], Leonardo Giordano Paterno[2] and Sônia N. Báo[1]*

[1]Department of Cell Biology, Institute of Biological Sciences, University of Brasília, Brasília/DF, Brazil; giovannalobo2012@gmail.com
[2]Laboratory of Research on Polymers and Nanomaterials, Institute of Chemistry, University of Brasília, Brasília/DF, Brazil; leonardopaterno@hotmail.com
*Correspondence: snbao@unb.br; Tel.: +55 61 99909-5005

Supplementary Fig. S1:

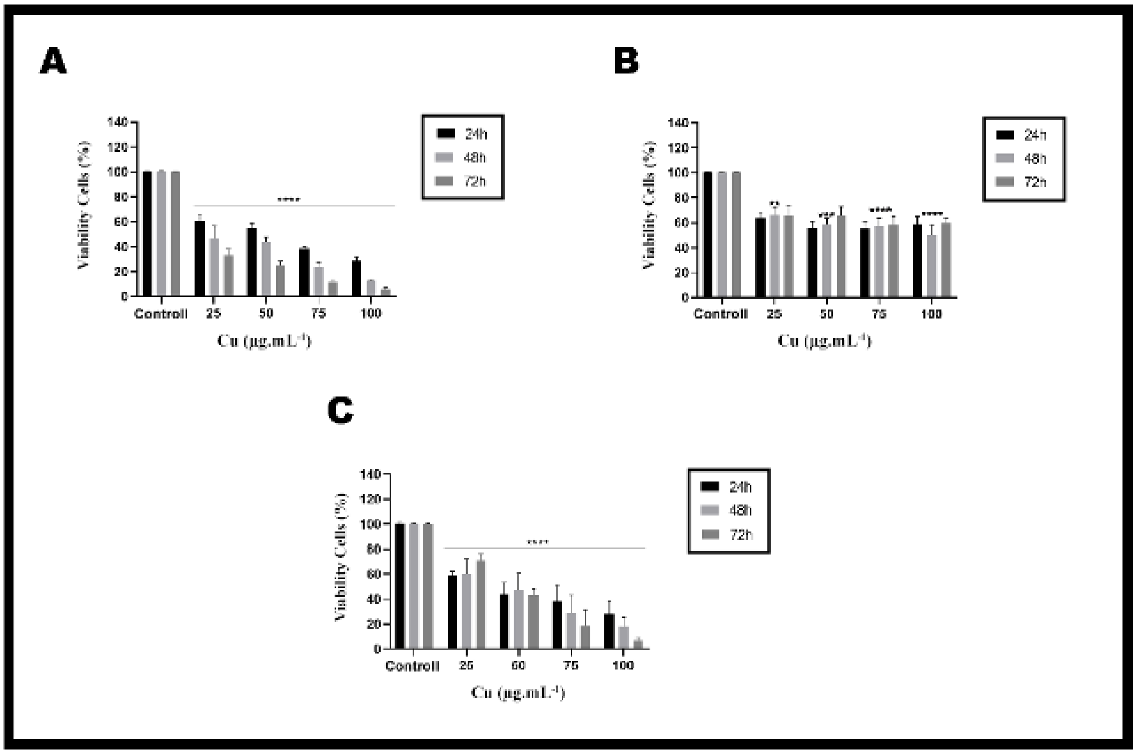

Figure S1: Evaluation of cell viability by alamarBlueTM of MCF7, MDA-MB-231, MCF 10A strains after 24, 48 and 72 hours of treatment with free Cu^{2+} at different concentrations. Viability chart of strain **(A)** MCF-7, **(B)** MDA-MB-231 and **(C)** MCF 10A. Bars represent percentage cell viability for each strain at the indicated concentrations. Data represent the mean ± SEM of three independent experiments in quadruplicates **$p < 0.01$, ***$p < 0.001$ and ****$p < 0.0001$. Treatment compared to untreated control.

Table S2: Inhibitory concentration of 50% free Cu^{2+} of MCF-7 and MCF 10A strains at 24, 48 and 72 hours.

Time	24 hours	48 hours	72 hours
MCF7	44.10 µg. mL^{-1}	42.97 µg. mL^{-1}	27.81 µg. mL^{-1}
MCF 10A	10.20 µg. mL^{-1}	12.46 µg. mL^{-1}	26.52 µg. mL^{-1}
